# Subinguinal Varicocelectomy With Loupes Only, Sans Surgical Microscope

**DOI:** 10.5812/numonthly.7588

**Published:** 2012-12-15

**Authors:** Petros Mirilas

**Affiliations:** 1 Centers for Surgical Anatomy and Technique, Emory University School of Medicine, Atlanta, USA

**Keywords:** Infertility, Varicocele

Dear Editor,

I would like to comment Abdelrahman and Eassa on their important paper on the assessment of loupe-assisted subinguinal varicocelectomy in infertile men (1).

Methodology was complete, including a group of patients and another of controls, of adequate sample size, defined with valid inclusion and exclusion criteria, and followed-up at 3 and 6 months with measures of seminal count/morphology and relevant hormones. 

As for surgical anatomy and technique, the authors correctly approached internal spermatic veins after opening the internal spermatic fascia. They correctly mention a ‘compartment of the vas’ internal to the internal spermatic fascia. Many experts, however, erroneously state that the vas sits outside the internal spermatic fascia (2). The vas indeed lies internal to the internal spermatic fascia (i.e. in the spermatic cord’s internal compartment) but is further ensheathed by a membranous layer continuing from the respective layer of the abdominopelvic extraperitoneal tissue. The internal spermatic vessels, nerves and lymphatics all within the internal compartment are similarly packed by another membranous sheath (3).

The authors preserved the veins of the vas, “except when abnormally engorged veins were evident”. My preference is not to ligate these, but instead cremasteric veins plus their anastomoses with the external pudendal vein. The cremasteric veins are found between the internal and external spermatic fasciae, i.e. in the cord’s external compartment. Intact veins of the vas serve for venous flow after ligation of the internal spermatic and cremasteric veins. Gubernacular veins were not ligated; consequently the testis was not delivered. I concur: if sacrificed, testicular venous return relies only on the small deferential veins (3).

With loupes only (sans surgical microscope), 0% recurrence and 0% hydrocele were achieved; admirable rates, totally comparable with those of subinguinal varicocelectomy under microscope -better than rates of others using loupes [2.9% hydrocele, 2.9% reccurence (4)]. Complication rates of subinguinal varicocelectomy without loupes— 15% hydrocele, 10% recurrence seem higher than others’ 7.3% hydrocele, 2.63% recurrence (5), which, however, merge inguinal and subinguinal procedures.

In statistical analysis, the authors unfortunately disregarded their paired and time-series design, and performed multiple, pair-wise comparisons, increasing probability of type I error. Instead, analysis should start with testing for normal distribution; if so, a 2-way-ANOVA for repeated measurements would reveal an overall difference; then post-hoc analysis should follow to detect differences between duos of data. In case of non-normal distribution, Friedman test should be applied for the time-series triplets of each group, and Mann-Whitney U-tests for duos of data at each time point (6).

In conclusion, the paper strongly supports loupe-assisted subinguinal varicocelectomy in infertile adults. It can be ameliorated in surgical anatomy regarding compartments of the spermatic cord, and has pitfalls in statistical analysis. Nevertheless, the paper has great virtues: excellent surgical results and justified semen and hormonal findings. What I liked most was the honest declaration ‘so it [this technique] should be used, especially in developing countries where the microsurgery equipment is not available every where”. I hope the authors obtain this equipment. I look forward to reading their next paper on microsurgical subinguinal varicocelectomy.
